# Massive metagenomic data analysis using abundance-based machine learning

**DOI:** 10.1186/s13062-019-0242-0

**Published:** 2019-08-01

**Authors:** Zachary N. Harris, Eliza Dhungel, Matthew Mosior, Tae-Hyuk Ahn

**Affiliations:** 1 Department of Biology, Saint Louis University, Saint Louis, MO 63103 USA; 2 Program in Bioinformatics and Computational Biology, Saint Louis University, Saint Louis, MO 63103 USA; 3 Department of Computer Science, Saint Louis University, Saint Louis, MO 63103 USA

**Keywords:** Metagenomics, Machine learning, Taxonomy profiling, MetaSUB, CAMDA

## Abstract

**Background:**

Metagenomics is the application of modern genomic techniques to investigate the members of a microbial community directly in their natural environments and is widely used in many studies to survey the communities of microbial organisms that live in diverse ecosystems. In order to understand the metagenomic profile of one of the densest interaction spaces for millions of people, the public transit system, the MetaSUB international Consortium has collected and sequenced metagenomes from subways of different cities across the world. In collaboration with CAMDA, MetaSUB has made the metagenomic samples from these cities available for an open challenge of data analysis including, but not limited in scope to, the identification of unknown samples.

**Results:**

To distinguish the metagenomic profiling among different cities and also predict unknown samples precisely based on the profiling, two different approaches are proposed using machine learning techniques; one is a read-based taxonomy profiling of each sample and prediction method, and the other is a reduced representation assembly-based method. Among various machine learning techniques tested, the random forest technique showed promising results as a suitable classifier for both approaches. Random forest models developed from read-based taxonomic profiling could achieve an accuracy of 91% with 95% confidence interval between 80 and 93%. The assembly-based random forest model prediction also reached 90% accuracy. However, both models achieved roughly the same accuracy on the testing test, whereby they both failed to predict the most abundant label.

**Conclusion:**

Our results suggest that both read-based and assembly-based approaches are powerful tools for the analysis of metagenomics data. Moreover, our results suggest that reduced representation assembly-based methods are able to simultaneous provide high-accuracy prediction on available data. Overall, we show that metagenomic samples can be traced back to their location with careful generation of features from the composition of microbes and utilizing existing machine learning algorithms. Proposed approaches show high accuracy of prediction, but require careful inspection before making any decisions due to sample noise or complexity.

**Reviewers:**

This article was reviewed by Eugene V. Koonin, Jing Zhou and Serghei Mangul.

**Electronic supplementary material:**

The online version of this article (10.1186/s13062-019-0242-0) contains supplementary material, which is available to authorized users.

## Background

While microbes make up a significant proportion of the biomass on the planet, their contributions to the function of most environments have only recently been explored. Starting in the 1980s with 16S rRNA profiling to metagenomic analyses today we have begun to probe how these microbial assemblages, the microbiome, shape their environments. Metagenomics, specifically, has fundamentally changed the way we think of the microbial landscape of countless biological and environmental spaces. From profiling soil communities [1, 2] to investigating the microbiome associated with human health and diseases [3], we can now explore how the microbiome creates harmony with other organisms in these spaces.

Metagenomic profiling has been particularly explored as a function of microbial impact on human health and diseases. This exploration exists as a function of direct analysis of human derived samples and samples of the human occupied environment. In 2007, the framework for the Human Microbiome Project (HMP) was set forward [3]. This project was a direct consequence of the Human Genome Project failing to account for the total function found to exist within the human body. The project sought to clearly define the concept of a core microbiome of healthy human participants while accounting for lifestyle, environment, physiology, etc. By 2012, after generating over 5000 samples and 3.5 terabasepairs (Tbp) of next-generation sequencing (NGS) data, the HMP identified trends in the structure of human microbiome, but also an incredible amount of diversity [4, 5]. This diversity stems from multiple backgrounds of human samples relative to phenotype, lifestyle, and country of origin [6–8]. Moreover, changes in the human microbiome have been associated with *Clostridioides difficile* infection [9–11], bacterial vaginosis [12–15], Parkinson’s disease [16], and potentially even commonplace challenges with mental health [17, 18].

As humans spend roughly 90% of their time indoors, the frequent association with microbial populations and human health has prompted deep exploration into the microbial landscape of the built environment [19]. Clear associations have been found in built environment-associated microbiomes as a function of ventilation, building purpose, and even within buildings as a function of room-purpose [20–24]. Of particular interest to human health is the microbiome of public transit systems, ever-increasing resources upon which millions of people rely every day. A recent analysis of New York City public transit systems showed a wealth of microbial data that is unable to be annotated as well as a microbial diversity that correlates with the diversity of the public transit users [25]. An analysis of the Hong Kong subway system showed that the airborne microbiome dynamically changes with human density [26]. These results often largely corroborate findings of human-derived samples that show high levels of diversity and that multiple factors explain the variance of the datasets.

With the increasing number of trends correlated with microbiome data is an increasing amount of data to be analyzed for any particular question. For example the HMP, as of 2012, had already generated nearly 3.5 Tbp of sequences after application of a quality control protocol from a total 8.8 Tbp that included human sequence removal, quality filtering and trimming of reads [4]. As of 2017, the second phase of the study (HMP1-II) increased the volume to over 24 Tbp [27] and total post analysis data could be a few times bigger than the sequences alone. It is only now becoming commonplace for labs to store that much data, but it is rare for labs to have the capacity to analyze that much data. In addition to the obvious challenge of metagenome assembly, there are increasing trends toward quantifying the total genomic content of a species (pan-genomes) [28], comparing disparate metagenomes, and even the functional analysis of those metagenomes. All of this brings forward an interesting computational challenge that has to be addressed moving forward. These computational challenges are a prime example of big data explorations in the biological sciences, a key interest of the committee on the Critical Assessment of Massive Data Analysis (CAMDA) [29]. In 2018, one of their major challenges is the construction and fingerprinting of a city-specific metagenome as characterized by the city’s subway system [30]. Here, we present our interpretation of that challenge.

Over the past decade, diverse metagenomics software tools have been developed for 16S analysis and shotgun metagenomic analysis [31]. Shotgun metagenomics data can be analyzed using several different approaches. The methodological approaches can be divided into two categories: read-based and assembly-based [32]. Read-based metagenomics analysis is useful for quantitative community profiling and identification of organisms especially if relevant references are available. MetaPhlAn2 [33] identifies clade-specific marker genes for evidence of the associated clade presence. This allows for rapid assignment relative to a small database as compared to a full database including many whole genomes and fast mapping aligner, Bowtie2 [34]. Nucleotide taxonomic classification tools including Kraken [35], Centrifuge [36], and Megan [37] are generally used for precise estimation of taxonomic abundances by aligning reads to *k*-mers or full reference genomes. Assembly-based workflows attempt to assemble the reads from one or more samples, group (bin) the contigs from these samples into genomes, then analyze the genes and contigs. Megahit [38], MetaSPAdes [39], and IDBA-UD [40] are the most widely used *k*-mer based assemblers for high-throughput NGS metagenomic data. Most metagenomic classification tools match reads or assembled contigs against a database of microbial genomes to identify the taxon of each sequence. Several strain-level resolution taxonomic profilers were recently developed [41–45].

There are few software tools providing the statistical methods and machine learning modules to derive microbiome-phenotype associations along with metagenomics-based prediction using taxonomic profiling. For example, MetAML [46] was developed for metagenomics-based prediction tasks and for quantitative assessment of the strength of potential microbiome-phenotype associations. Reiman et al. [47] explored convolutional neural network to predict of the phenotype of a genomic sample based on its microbial taxonomic abundance profile. Additionally, VirFinder [48] was developed for virus contig identification with a *k*-mer frequency-based machine learning model from metagenome assemblies. However, they all vary from the goal of our work which is to compare two widely-used methodological approaches, read-based and assembly-based, for metagenomics researches with multiple machine learning methods with a focus on extremely large data sets.

In this paper, we present two approaches using various machine learning techniques. First, we propose a read-based taxonomy profiling and prediction method. Both genus and species level information are explored as machine learning features and used for prediction from individual metagenomic profiling of samples. Second, we investigate a reduced-representation assembly-based machine learning prediction method. From various experiments using diverse machine learning techniques in the two proposed approaches, the Random Forest (RF) technique outperforms other machine learning techniques with a higher level of accuracy.

## Methods

### Data sets

CAMDA delegates received access to hundreds of novel MetaSUB samples, comprising several hundred gigabasepairs (Gbp) of whole genome shotgun (WGS) metagenomics data. Samples were collected from multiple surfaces in mass-transit systems (handrails, ticket machines screens and keypads, plastic, metal, wooden benches, etc.). The primary data set covered multiple cities around the world, with tens of samples per city. The info of samples of eight different cities are provided in Table 1. Together, they form a unique resource for the study of biodiversity within and across geographic locations or surface types.

**Table 1:** Primary and unknown data sets. Sample size for different cities and unknown, along with clean files (size is in GB)

Location	Acronym	Number of samples	Total size (GB) of clean files (FASTQ format)	Total number of reads (filtered)
Auckland, New Zealand	AKL	15	47.8	136,022,160
Hamilton, Canada	HAM	16	61.5	179,554,428
Sacramento, US	SAC	16	36.5	105,326,430
Santiago, Chile	SCL	20	215.3	613,721,390
Offa, Nigeria	OFA	20	438.2	1,267,427,220
Porto, Portugal	PXO	60	132.2	380,372,340
Tokyo, Japan	TOK	20	308.6	1,103,076,136
New York, US	NYC	26	368.8	1,086,713,476
Unknown	UNK	30	75.3	219,935,058

In addition to the primary data set, complementary independent data sets were provided for exploration. In our analysis, we focused on the presentation of 30 new samples that accompanied the goal of predicting the city of origin. Throughout our analysis we refer to this set as the ‘the test set’ or ‘the unknown data set’. The challenge also provided two other questions, not addressed here, about ‘mystery’ cities not featured in the primary data set. The number of samples and sequence sizes of that primary data set are described in Table 1.

### Computing facilities

We performed the large scale analyses using in-house computing facilities. One workstation (Intel Xeon E5–2640 v3 2.6GHz 16 cores 32 threads, 128GB RAM, 50 TB disk), one small cluster (3 nodes, each node has 24 cores 48 threads with 2 X Intel Xeon E5–2650 v4 2.2GHz and 256GB memory, 50 TB disk), and a university computer cluster consisting of 100 compute nodes, the 20 newest of which contain Intel Xeon E5–2690 v3 @ 2.60GHz processors. We especially used high memory nodes with 512GB of RAM, 117 TB InfiniBand connected network storage, and Infiniband interconnection of nodes.

### Sample preprocessing

BBDuk of the BBTools suite [49], designed for filtering or trimming reads for adapters and contaminants using *k*-mers, was used for quality filtering and for the removal of potential adapter contamination from all the samples. Specifically, reads were trimmed for quality from both the right and left termini (option: qtrim = rl) at a quality threshold of Q10 (option: trimq = 10). Adapters were removed based on the precompiled list of adapters in BBDuk.

### Approach

**Fig. 1:**
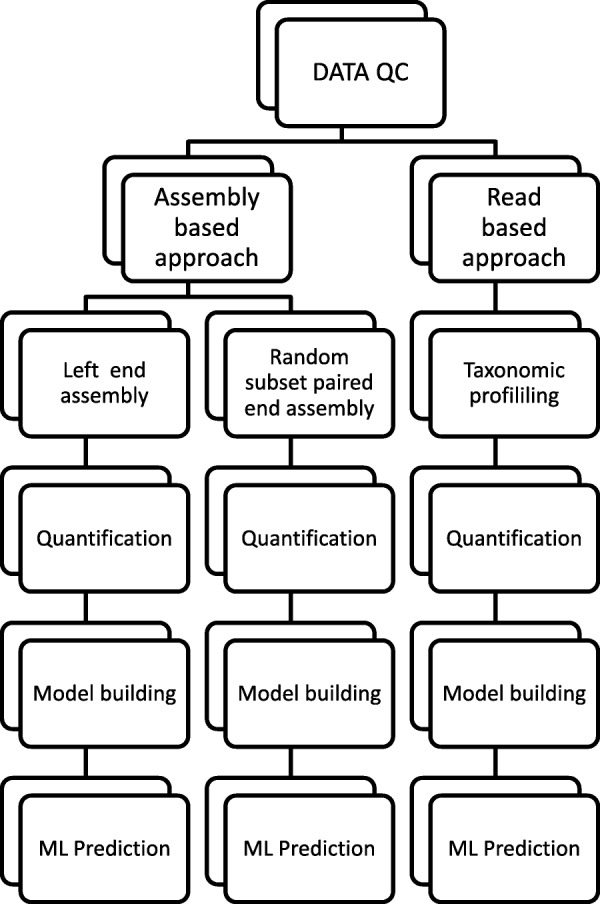
The analysis pipeline presented in this paper. Here we show the two-pronged approach used in this analysis. The data were analyzed under a read-based and assembly-based approach. In the read-based approach, we used taxonomic profiling for the generation of machine learning features for city prediction. In the assembly-based approach, we used two different reduced representation paradigms to generate features for machine learning features

In order to efficiently handle the magnitude of data required for this analysis, we opted to explore these data using two major approaches that greatly reduce the computational load of analyses at any given time: one is a read-based taxonomy profiling and quantification, and the other is a metagenome assembly-based approach as shown in Fig. 1. For each of these approaches, we generated abundances of the microbial species (or proxies thereof) for the use in machine learning-based predictions.

### Read-based taxonomic profiling and quantification

Read-based metagenomic profiles were obtained for the preprocessed samples using MetaPhlAn2 [33]. We note, that while some interpretations of MetaPhlAn2 include limited sensitivity especially on the case of similar genomes presenting in a sample [50], we have included it in this analysis for precisely that reason - it limits the potential search space and fast for taxonomic profiling by the marker-gene database. We executed each iteration of MetaPhlAn2 using 16 cores. The metagenomic profile and the estimate of the number of the reads in each clade obtained after running MetaPhlAn2 were extracted from each output file using custom script and the number of reads in each clade was merged into a table using the MetaPhlAn2 utility script. From the merged table, species and genus level information was extracted and used for building the machine learning model.

### Metagenome assembly and quantification

For the assembly-based metagenomic analysis, we further divided the work into two analysis paradigms to ease the computational necessity of the analysis. These paradigms are summarized in Additional file 1: Fig. S1, where the paradigm PP (the paired end paradigm) extracted a random set of all reads while maintaining the paired end structure of the data, and PL (the left-only paradigm) used only the left reads from each sample. After extraction of these reads, Megahit [38] was used to assemble the reads in each of the two paradigms with default assembly parameters on a university cluster node with 512 GB of RAM. Megahit was allowed access to all of that memory (option: --mem-flag 2) and a verbose output was written (option: --verbose). The abundance of each generated sequence was estimated for all paired-end reads with BBMap, a short-read aligner for DNA and RNA-seq data of BBTools [49], and each set of sequences was filtered such that only long sequences were retained, but the mapping rate of both assemblies was roughly equal (Additional file 2: Figure S2). This meant that PP was filtered for sequences longer than 5000 bp and PL was filtered for sequences longer than 1000 bp.

### Machine learning and city prediction

To analyze large scale and complex biological data sets effectively, we notice an increasing use of machine learning techniques. Based on prior work, we analyzed each of the approaches using two major algorithms: linear discriminant analysis (LDA) and random forests (RF). LDA is a supervised classification technique proposed for dimensionality reduction to project the features in higher dimension space onto a lower dimensional space. RF is a scheme of ensemble-based decision trees with a combination of tree predictors where each tree in the ensemble is grown correspondingly with a random subset of features. We selected LDA and RF to compare parametric (LDA) vs nonparametric (RF) machine learning techniques. In the areas of biomedical science and bioinformatics, the LDA and RF are popular choices for efficiency and accuracy. Support vector machines (SVM) and multi-layer perceptrons (MLP) are also tested for benchmark to the RF.

In each approach, the abundances (either derived from MetaPhlAn2 for read-based or BBMap for assembly-based) were used as features for city-based predictions. Machine learning analyses were conducted using Scikit-Learn [51] and caret R-package [52] - both of which are popular implementations of common machine learning algorithms in Python and R respectively. For the LDA, default parameters were used. For the RF, 50 random decision trees were used in the following naïve hyperparameter searching through cross validation (Additional file 3: Figure S3). For each analysis, the metric of interest was the accuracy of prediction (Accuracy = (TP + TN)/(TP + TN + FP + FN)) and this metric is presented in two ways: 1) a 10-fold cross validation accuracy and 2) the performance on 30 samples held out by CAMDA. For 10-fold cross validation accuracies, the data were randomly split in ten train/test partitions, and the final prediction were made using a model trained on all available samples.

## Results

### Read-based machine learning prediction

For the fast turnaround time of running MetaPhlAn2 with 223 primary data set from eight cities, we used both multi-threaded option provided in MetaPhlAn2 and multi-job submission script to run the MetaPhlAn2 jobs in parallel in our many-node cluster. Then, we merged each sample taxonomic profile into one large table. The merged table has four kingdoms, 17 phyla, 33 classes, 59 orders, 160 families, 353 genera, and 865 species, and the relative abundance of each was quantified. We first evaluated the prediction accuracy using the primary data set after splitting the data set into ten randomly generated 70/30 training/test partitions. To generate model training features, we tested both genus-level taxonomy profile and species-level taxonomy profile. In short, species-level model predictions outperformed that of the genus-level. Below we report results from the species-level prediction.

**Fig. 2:**
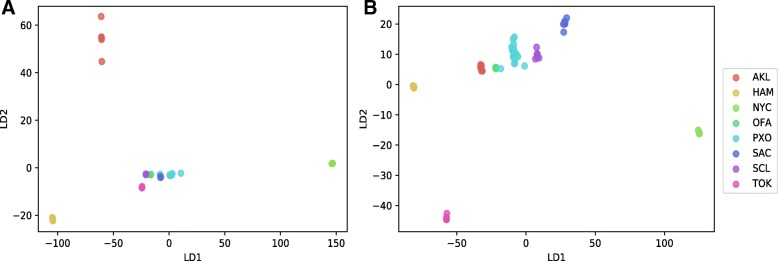
LDA plots of the read-based approach. **a** LDA with all species. **b** LDA with rare species (present in < 5% of samples) removed

**Fig. 3:**
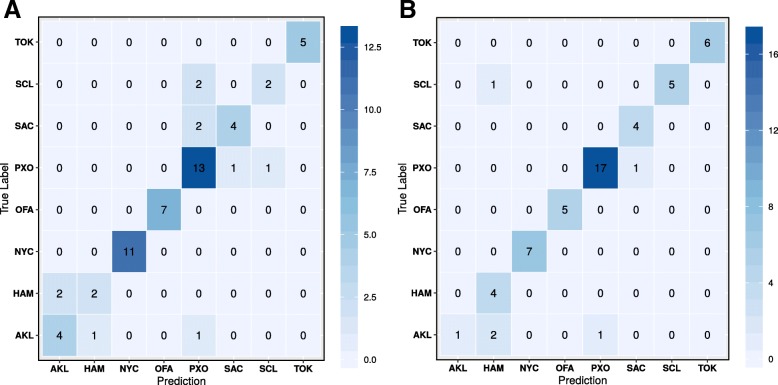
Confusion matrices for the read-based approach. **a** Confusion matrix for the random forest model trained on a random 70/30 train/test data partition. **b** Confusion matrix for the random forest model trained on a random 70/30 train/test data partition of the rare-species-removed data set

We investigated linear discriminant analysis (LDA) and random forest (RF) machine learning techniques. Based on species-level LDA, the samples from each city displayed very little variance (Fig. 2a), but the model had a very low prediction accuracy (~ 20%). Like the principal component analysis (PCA) dimension reduction approach, the LD scatter plot using the 1st two discriminant dimensions can show the supervised clustering of each group. The LDA model was tested again after removing the rare species where the abundances of species present in < 5% of samples. The rare-species-removed LDA experiment shows much better separation of cities (Fig. 2b), but the model prediction was still very low (22.08% accuracy range of 9.52–43.85%). To try to improve the model performance, we examined the RF model using default parameters. The ten-fold 70/30 train/test partitions were able to achieve a mean accuracy 83% (Fig. 3a, for example) accuracy with 95% confidence interval between 70 and 91%. Figure 3a shows the confusion matrix that is a technique for summarizing the performance of a classification algorithm. Because classification accuracy alone can be misleading if there are an unequal number of observations in each class or more than two classes in the data set, calculating a confusion matrix can provide a better idea of what the classification model is getting right and what types of errors it is making. In machine learning classification problems, an imbalance of the frequencies (e.g., sample size) of the observed classes can have a significant negative impact on model fitting. One technique to resolve such a class imbalance is to subsample the training data in a manner that mitigates the issues. Using the subsample technique optimization, we increased the accuracy of prediction to 91% with 95% confidence interval of 80–93% (Fig. 3b). To compare approximate system usage and elapsed time for read-based and assembly-based analyses, we used one-node based calculation in Table 2. The wall-clock time using read-based approach can be reduced and near linearly scaled if multi-node cluster is available.

**Table 2:** The system usage for read-based approach and two (PP and PL) assembly-based approaches (1 node based calculation)

Method	CPU usage	Wall Clock Time (Hours)	Memory Usage
Read-based	16 cores	187.2	62 GB of RAM
PP Assembly	24 cores	83.28	500 GB of RAM
PL Assembly	24 cores	38.4	500 GB of RAM

After we exhaustively validated model performance in our assigned training data set, we used the entire assigned data set as training data set to predict and assigned 30 unknown samples (Table 3). Based on the provided true labels from CAMDA, Table 3 shows that the read-based RF model correctly identified 18 out of 30 samples. 10 out of 12 false predicted samples are from New York city. The accuracy rate is lower than primary data set prediction by the New York city samples, but the read-based RF approach shows good prediction in most of other cities.

**Table 3:**
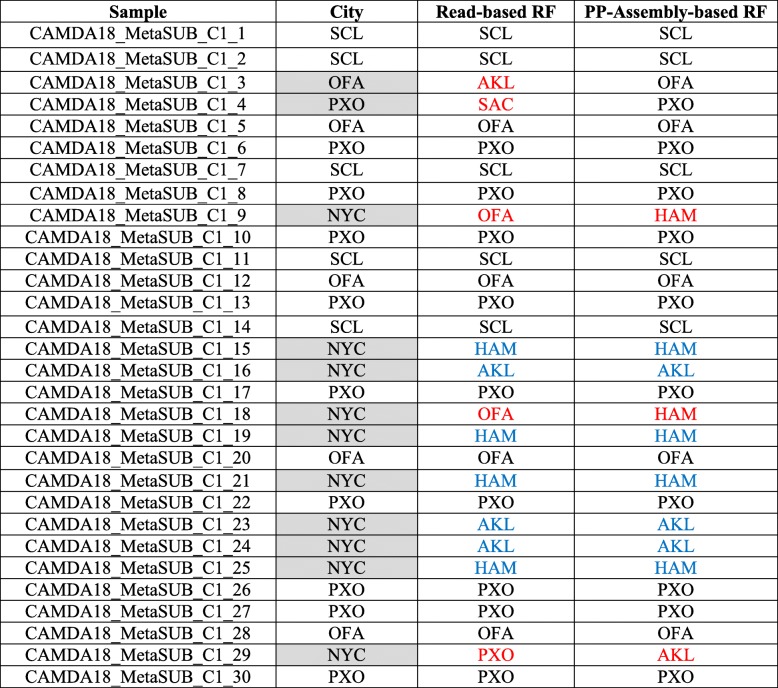
The evaluation of 30 unknown cities prediction from read-based RF and PP-assembly-based RF. The predictions that do not match true labels, and do not match between two predictions are shown in red. The predictions that do not match true labels, but match between two predictions are shown in blue

### Assembly-based machine learning prediction

In order to efficiently handle the magnitude of data required for this analysis, we additionally opted to use a reduced-representation assembly-based methodology. This has been achieved using two different paradigms: PL represents a metagenome assembly using only the left reads from all samples and PP stands for a paired-end assembly using only a random even subset from all cities. The PL approach was hypothetically more computationally efficient without considering paired-end information in the assembly program, but the PP should have generated higher quality sequences. As we expected PP generated many more longer sequences. To test different scenarios, we used PP assembled length > 5000 bp (242,348 assembled sequences) and PL assembled length > 1000 bp (2,070,675 assembled sequences) for training features which minimized the number of features for computation, but approximately normalized the mapping rates of the raw reads back to the assembly (Additional file 2: Figure S2).

**Fig. 4:**
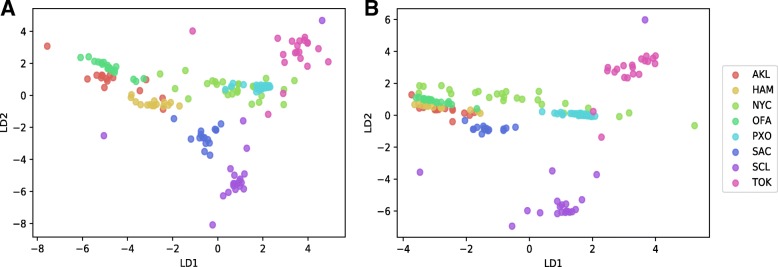
LDA of the assembly-based approach. **a** LDA of the random paired-end subset assembly (PP). **b** LDA of the left-only subset assembly (PL)

**Fig. 5:**
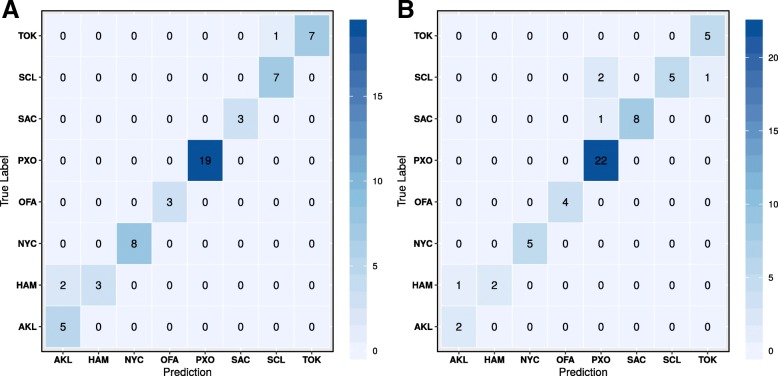
Confusion matrices for the assembly-based approach. **a** Confusion matrix for the random forest model trained on a random 70/30 train/test data partition in the random paired-end subset assembly. **b** Confusion matrix for the random forest model trained on a random 70/30 train/test data partition of the left-only assembly

As the read-based experiments, we explored LDA and RF machine learning techniques using ten 70/30 train/test partitions of the primary data set. While the separation was not as clear as the rare-species removed model in the read-based approach, the PP-based model did achieve an accuracy of 71.8% (57.1–93.8%) (Fig. 4a) Using a random forest the accuracy improved considerably at 88.5% (76.4–95.2%) as shown in Fig. 5a. For the PL-approach, results were very similar with the linear discriminant analysis showing an accuracy of 69.3% (58.5–82.4) (Fig. 4b) and the random forest showing an accuracy of 89.7% (64.7–100%) (Fig. 5b). To put these results in a broader context, we tested other commonly used models in bioinformatics including the support vector machine (SVM; default params) and the multi-layer perceptron (MLP) using the PP paradigm. SVM models were tested using both normalized (SVM-N) and non-normalized (SVM) data, and the MLP models were tested using both default nodal architectures (1X100; MLP) and a more complex nodal architecture [((4X256) + (4X128) + (4X32) + (8X16)); MLP-C]. These models consistently performed poorly using the PP paradigm (Table 4), so they were not explored in the larger PL paradigm.

**Table 4:** Model prediction accuracies based on cross-validation of the training set. RF-10: Random forest with 10 random decision trees, RF-20: Random forest with 20 random decision trees, SVM: default support vector machine, SVM-N: SVM with normalized features, MLP: default Multilayer perceptron, MLP-C: Multilayer perceptron with complex nodal architecture (described in methods)

Model	Accuracy
RF-10	87.9
RF-20	89.7
SVM	43.1
SVM-N	32.8
MLP	63.7
MLP-C	55.2

After we completed the experiments of prediction of the primary data set, we used the assembly sequences as features of a training data set to predict unknown 30 samples. Based on the provided true labels from CAMDA, Table 3 shows that the assembly-based RF model accurately predicted all cities except New York city. This approach correctly identified 20 out of 30 samples without the 10 samples from New York City. The assembly-based and the read-based results show very comparable and related predictions.

## Discussion

The data presented in the CAMDA challenge offer a unique ability to identify methods of appropriate analysis for large and noisy metagenomic data sets. Here we proposed two different approaches to collect features from the same city samples to utilize them for unknown sample prediction using machine learning techniques. The first approach is a read-based taxonomy profiling and prediction method. The second approach is an assembly-based profiling and prediction technique. Although the final random forest prediction results for both approaches show very similar accuracies, the two approaches have significant differences especially in system usage. As CAMDA focuses on exploring and solving big data challenged in life science using advanced and modernistic ideas, it is worthy to describe the design concept of two proposed approaches and their benefits and detriments as they apply to massive-scale metagenomic data analysis.

Overall, our results indicate that while both of our approaches have different advantages and drawbacks, they provided very similar results when it comes to the final analysis. More specifically, even though the approaches are different, they both underperformed in the prediction of one specific city label, NYC. The differences in the approaches indicate that this performance is most likely outside the purview of the approaches themselves. Most likely, samples were taken from a variety of surfaces that could foster different microbial taxa and the full extent of that space may have been unavailable in the initial training data. Interestingly, our results may have broader implications. Namely, our results indicate that read-based profiling is functionally equivalent, and in fact slightly worse when looking to the test set, than essentially throwing away half of the available data for the assembly-based protocols. While this result is theoretically reasonable as our taxonomy-based approach should lower sensitivity, the scope of this finding is substantial and favors the use of metagenomic assembly-based protocols. The remainder of this discussion should serve to guide biologists to make appropriate decisions for analyzing large metagenomics data sets under variable circumstances and their questions.

The first read-based approach is good for users who do not have large-memory system. In here, we used MetaPhlAn2 for each sample profiling. MetaPhlAn2 or other read mapping based software tools usually do not use high-memory for one sample analysis. For example, MEGAN [37], a widely used taxonomy profiling algorithm with read mapping, usually uses ~5X the memory of the sample size depending on algorithm selection (for example, the weighted LCA algorithm uses higher memory than the LCA algorithm). MEGAN-LR [53], a newer LCA-based algorithm for taxonomic binning, also uses desktop level memory on the scale of tens of GB per sample. Most alignment-based metagenomic profiling tools use fast and memory efficient aligners such as Bowtie2 [34], BWA [54], and LAST [55]. The user, however, should consider running time. Aligning and profiling of one metagenomic sample is not that long, but if you have thousands of samples, it will take roughly thousands of times of each sample run time. If user can access a multi-node cluster, batch job scripts or simple message-passing-interface (MPI) programs can reduce the wall-clock time dramatically.

The second assembly-based approach is an appropriate method for users who can access large memory computing resources. Although there are few scalable de-novo metagenome assembly programs (such as Ray Meta [56]) available, most metagenome assembly programs require very large memory (10X of sample size) for the large-scale merged data set. Here, we showed that reduced-representation subset of the total data set also can derive precise prediction when used in conjunction with machine learning. We showed that this was a valid approach using two different assembly-based paradigms. First, we showed that a random subset of paired end reads (PP) were sufficient to predict the correct city label. This approach is especially useful for researchers who have access to large computational resources but may be time limited. Subsetting the data requires only a fraction of the time for assembly. Second, we showed that the left-only paradigm (PL) performed just as well as the random subset of paired end reads. This result is especially useful in time-limited systems as the assembly takes roughly half the time of the of the PP-based subset. Here, we do warn users that paired-end data tend to generate better (less fragmented) assemblies. The fragmentation of the PL method meant that more sequences were required to generate the same mapping rates as the PP method. The meant that the resultant ML models had ~10X as many features. This meant that models like LDA and RF took longer (albeit on the scale of minutes), but larger models like multi-layer perceptrons with complex nodal architectures took too long to consider in the scope of this manuscript.

While the topic of biological interpretation of these data are beyond the scope of this analysis, many researches will likely include biological interpretation downstream in their analysis. The read-based approach, shown here with MetaPhlAn2 is an excellent choice for these analyses. Inherent in the execution of MetaPhlAn2, the data are placed in a biological context. Users would be able to see how different bacterial families, genera, or species compare within and between samples. This is also possible in the assembly-based approach, but requires even more computationally intensive analyses. For example, the metagenomes can be binned using alignment based binning tools [57–60], and the binned metagenomes could be taxonomically assigned using SendSketch [49] or BLAST [61]. Additionally, the different approaches could be combined, and the metagenomes can be fed to community profiling tools like MetaPhlAn2 for biological interpretation.

## Conclusions

For the last decade, a cultivation-independent metagenomics approach, in which all microorganisms in a sample are directly sequenced together, has been intensely applied to understand microbes’ impact on human health, plant, soil, water, and so on. A new generation of sequencing technologies accelerated research, but left a vast amount of metagenomic sequencing data to be analyzed. Software and high-performance computing systems that could speed analysis are still lacking. It is important to develop novel computational algorithms or pipelines to decipher terabytes of metagenomic sequencing data quickly and precisely. We here proposed two approaches to analyze the large-scale data set efficiently: one is read-based profiling approach and the other is reduced data set assembly-based approach. Multiple machine learning techniques were investigated and incorporated in the pipeline to predict unknown samples precisely. Overall, these approaches shows promise although more dedicated work is required to increase the prediction accuracy.

## Reviewers’ comments

### Reviewer’s report 1 - Eugene V. Koonin

**Reviewer comments:** The authors present two machine learning techniques to analyze metagenomic data. I believe that the methods are sound and could be useful to many researchers working with metagenomes. The authors explicitly indicate that biological interpretation is beyond the scope of the present work and briefly discuss the directions for extending their methods into the biological domain. This approach somewhat limits the impact of the article but is fully legitimate. Within the limitations mentioned above, I do not see significant flaws in the article.

**Author’s response:** *The authors would like to thank you for your time and effort to review our paper. The comments are greatly appreciated.*

### Reviewer’s report 2 - Jing Zhou

**Reviewer comments:** In this paper, the authors explored different abundance-based machine learning methods to predict city identity based on its subway metagenome. They examined two different approaches to generate metagenomic profiles – one is sample-based taxonomy profiling and the other one is reduced-representation assembly-based method. They found the Random Forest (RF) machine learning method yielded highest prediction accuracy (i.e. 91%) among other machine learning methods. For an independent testing set, the RF method with sample-based taxonomy profiling method correctly identified 18/30 samples. Although both profiling methods have shown very similar accuracy using RF methods, the authors pointed out the two methods have different requirement in system usage and provided recommendation for different systems. This information would be very useful, when it comes to choose profiling methods and prediction methods. I believe this paper fit the standard of Biology Direct and should publish with the following comments addressed.

**Author’s response:** *The authors would like to thank you for your time and effort to review our paper. The comments are greatly appreciated.*

**Reviewer comments:** Major Comments: 1) In the background session, I would expect the authors provide more background on the methods they used in the paper—especially the profiling methods.

**Author’s response:** *We agree that the methodology of our approaches should have been more explicitly stated in the “Background” section. As such, we have amended out “Background” section to include this level of detail.*

**Reviewer comments:** 2) Also, is there any other paper has used a similar combination of genomic profiling and machine learning methods? If there is any, how the results compared to the study here?

**Author’s response:** *To address this, we included a paragraph in the “Background” section.*

**Reviewer comments:** 3) I wonder if surfaces information is also available in the data set. If so, is that possible to use the best approach used in this paper to predict city identity+ surface identity? It may beyond the scope of this paper, but it would be an interesting question to explore in the future.

**Author’s response:** *This is an excellent comment. Unfortunately, we were not provided with the surface information for all of the samples through the CAMDA challenge. As such, we are unable to adequately analyze these data in that light. However, we absolutely agree that this would be a great comment to explore in the future in CAMDA challenges.*

**Reviewer comments:** Minor Comments: 1) The conclusions in the abstract did not provide any useful information to the readers. The main findings in the paper should be emphasized 2) The authors should provide the prediction accuracy for the independent testing set in the abstract as well. 3) In the method part, I think they should move the second paragraph to introduction. Also, it is confusing to me, how did the authors know which 30 were new samples? It states in the paper “About 30 new samples from different cities and surface types already featured in the primary dataset- can you tell which?”

**Author’s response:** *We have updated the “Results” and “Conclusions” paragraphs in the “Abstract”. “Data sets” subsection in the “Methods” section has been amended to more clearly describe our approaches to the specific challenge.*

### Reviewer’s report 3 - Serghei Mangul

**Reviewer comments:** Major comments: The caption to the figures are missing and need to be added More details of sequencing datasets need to be provided. For example, read the length of each dataset (Table 1).

**Author’s response:** *The authors would like to thank you for your time and effort to review our paper. The comments are greatly appreciated. We would like to kindly point that the captions of figures were provided in the main manuscript prior to the References section called “Figure Descriptions:” after following Biology Direct journal submission guidelines about figures. As reviewer commented, a column with read information has been added to* Table 1*.*

**Reviewer comments:** According to a recent benchmarking paper, Metahplan2 suffers from low sensitivity: Sczyrba, Alexander, et al. “Critical assessment of metagenome interpretation—a benchmark of metagenomics software.”; Nature methods 14.11 (2017): 1063. Authors need to comment on these issues with Metahplan2 and warn the users about this.

**Author’s response:** *We agree that MetaPhlAn2.0 could have low sensitivity especially in the case of closely-related genomes coexisting in the samples. That is why several strain-level resolution taxonomic profilers were recently published including Sigma* [45]*, that we developed before, ConStrains* [44]*, MIDAS* [43]*, StrainPhlAn* [41]*, and StrainEst* [42]*. However, most strain-level resolution profilers are computationally expensive and requiring large reference database with many genomes. In the CAMI manuscript, the authors stated that “In terms of precision, MetaPhlAn 2.0 and “Common Kmers” demonstrated an overall superior performance, indicating that these two are best at only predicting organisms that are actually present in a given sample and …*” *. In addition, MetaPhlAn2 allows very fast assignment by the smaller marker gene and fast mapping aligner, Bowtie2 that has a great fit into this massive metagenomic analysis. That is why we selected MetaPhlAn2 for our massive data analysis, and the results showed good accuracy from it. Based on reviewer’s comment, we added sentences in the “Read-based taxonomic profiling and quantification” subsection in “Methods”.*

**Reviewer comments:** P 7.line 162. Details of the packages used needs to be explained. What exactly they do?

**Author’s response:** *The sentences about machine learning library have been updated.*

**Reviewer comments:** Line 176. Data were divided into training and test partitions. The validation datasets need to be added. Ideally from a different cohort or from the same one. If this is impossible, the authors need to clearly provide reasoning.

**Author’s response:** *This is a very valid criticism of our manuscript. For this analysis, we opted not to include a validation set so as to maximize the volume of data available to train the models. We contend that, as this is a purely theoretical exercise not to be used for actual model deployments, this deviation from expected protocols is justified. We hold this to be true for two major reasons: 1) the data are highly imbalanced and 2) we have relatively few samples. This could then give us a very biased interpretation of our results. Using our method, we set aside the initial test set and then estimated model performance using different random partitions of the available training data (comprehensive cross validation). Perhaps, our most egregious deviation from expected protocols was attempting to tune the random forest hyperparameter (n_estimators in SciKit Learn) within this framework. In our approach, we simply used a relaxed implementation of the bootstrapping to iterate over several random cross-validation splits to find an appropriate range (Efron and Gong 1983). We have clarified out language to describe this throughout multiple section of the manuscript.*

**Reviewer comments:** The paper suggests that the prediction accuracy was 20%. Page 8. Line 182. How the prediction accuracy was calculated? This needs to be added to the paper.

**Author’s response:** *In the “Machine learning and city prediction” subsection in “Methods” section, we have amended the manuscript methods to include a definition of accuracy.*

**Reviewer comments:** Line 201/ page 9. The paper claims that many NYC sample failed to be identified. The immediate reason can be that NY is low coverage samples (> 2 M reads). The authors need to further investigate this and adjust for total coverage if this is was not done before. One approach is to subsample all samples to the same coverage (number of reads). Also was the read length of NY different from the rest?

**Author’s response:** *The reviewer outlines several really good potential explainers of our inability to appropriately predict the NY samples. Unfortunately, they are probably no closer than what we could come up with. As we added a column to* Table 1*, NY is the third largest sample. As our models are relative-abundance based, we opted not to adjust for coverage. This was primarily because we could not have applied the same filters to the testing set.*

**Reviewer comments:** The figure comparing marker gene-based approach (Metahplan2) and assembly one (Megahit) needs to be added. Maybe with the best classifier. This will help the reader better understand the difference between those approached.

**Author’s response:** Table 3 *shows the evaluation of 30 unknown cities prediction from read-based RF and PP-assembly-based RF to compare the power of two approaches.* Figures 3 and 5 *also show confusion matrices of training dataset for the read-based approach and the assembly-based approach.*

**Reviewer comments:** P 11. Line 257. Both marker gene-based approach (Metahplan2) and assembly one (Megahit) show similar results. The interpretation if this needs to be added to the Discussion section. Why low sensitivity of Metahplan2 does not affect the results.

**Author’s response:** *We have added a paragraph to the “Discussion” section addressing this issue and discussing our results overall.*

**Reviewer comments:** Minor comments: The paper mentioned the association of microbiome with mental health. The authors are recommended to add an additional citation supporting the association of microbiome with mental health: Loohuis, Loes M. Olde, et al. “Transcriptome analysis in whole blood reveals increased microbial diversity in schizophrenia.” Translational psychiatry 8.1 (2018): 96. P 3 line 75.

**Author’s response:** *Thank you for providing the reference paper. We have amended the citation for this section to include this work and a couple more recent analyses of similar approached.*

**Reviewer comments:** The paper claims that post analysis is at least a few times bigger than the sequencing data. This is unexpected and needs to be clarified with supporting results or reference.

**Author’s response:** *In most bioinformatics researches, it is naturally common to keep intermediate processed files with original sequence files for possible secondary analyses or any other purposes. Therefore, it will be safe for researchers to prepare few times larger available storage than amount of sequencing data size to analyze the data, but it is not always true as reviewer commended. By following of reviewer’s comment, we modified the sentence.*

**Reviewer comments:** P 4. Line 77. Definition of pan-genomes needs to be provided.

**Author’s response:** *We have updated the paragraph.*

## Additional files

Additional file 1: **Figure S1.** A schematic view of the reduced-representation paradigms for the assembly-based approach. In the random paired-end subset (PP), half of each city was extracted randomly while maintaining the paired-end structure of the data. In the left-only subset (PL), only the left read from each sample were used for the assembly. (PDF 656 kb)

Additional file 2: **Figure S2.** Mapping rates of the cleaned reads back to the metagenome assembly. The random paired-end subset (PP) assembly is shown in red. The left-only subset (PL) assembly is shown in green. (PDF 5 kb)

Additional file 3: **Figure S3.** Hyperparameter tuning for n_estimators in the assembly-based approach. Each figure shows accuracy results from a series of random decision tree constructions and random train/test partitions for each of those constructions. (A) Hyperparameter tuning of the random paired-end subset assembly (PP). (B). Hyperparameter tuning of the left-only assembly (PL). Note: The difference is point count is from fewer tests in the PL assembly as it had 10X as many features and took much longer to train and test. (PDF 2103 kb)
