# Supplementary figures and images for: Massive metagenomic data analysis using abundance-based machine learning
Source: Biol Direct. 2019 Aug 1;14:12. doi: 10.1186/s13062-019-0242-0 (PMC6676585; doi:10.1186/s13062-019-0242-0)

PP:

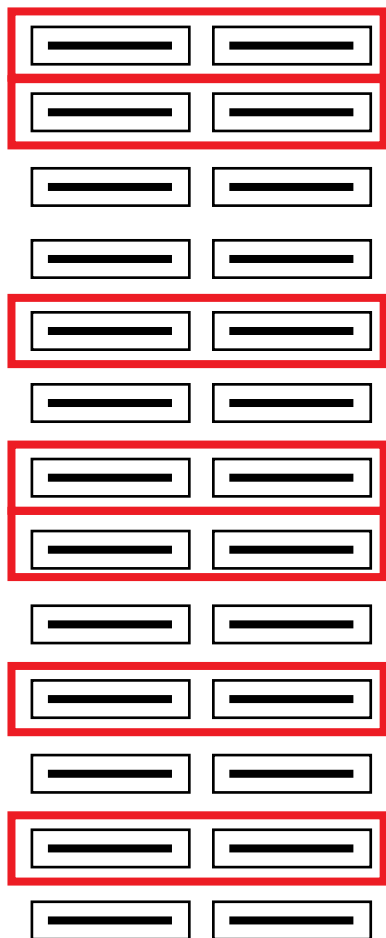

PL:

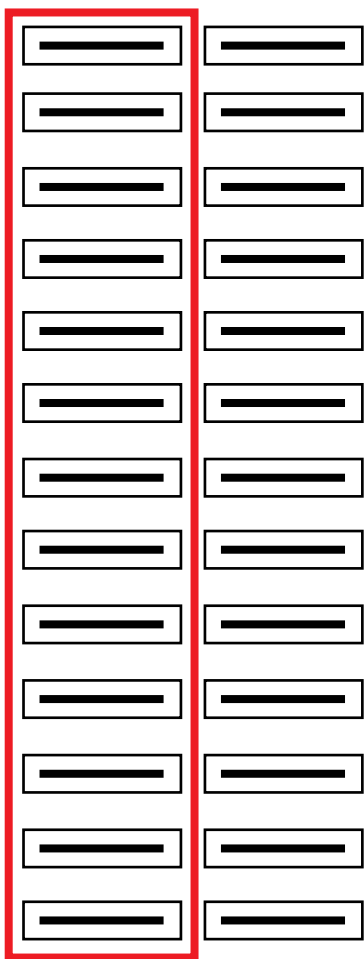

Supplement: Supplementary file 1 — Figure S1. A schematic view of the reduced-representation paradigms for the assembly-based approach. In the random paired-end subset (PP), half of each city was extracted randomly while maintaining the paired-end structure of the data. In the left-only subset (PL), only the left read from each sample were used for the assembly. (PDF 656 kb) [file 13062_2019_242_MOESM1_ESM.pdf]

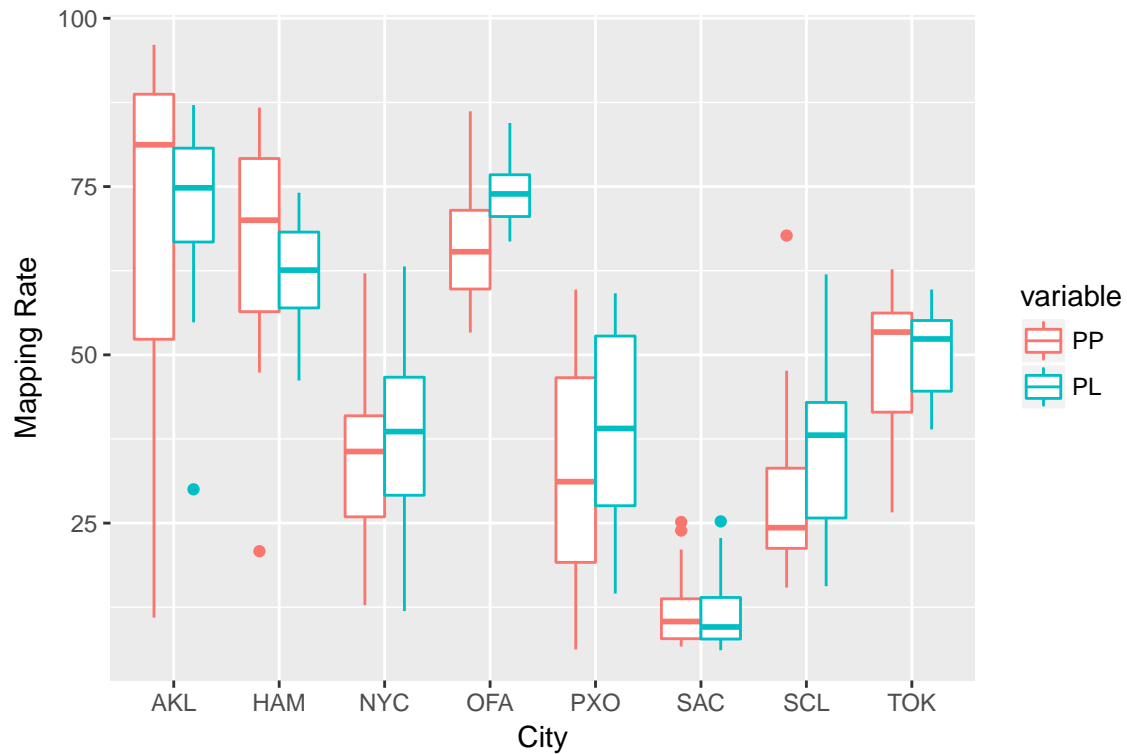

Supplement: Supplementary file 2 — Figure S2. Mapping rates of the cleaned reads back to the metagenome assembly. The random paired-end subset (PP) assembly is shown in red. The left-only subset (PL) assembly is shown in green. (PDF 5 kb) [file 13062_2019_242_MOESM2_ESM.pdf]

A

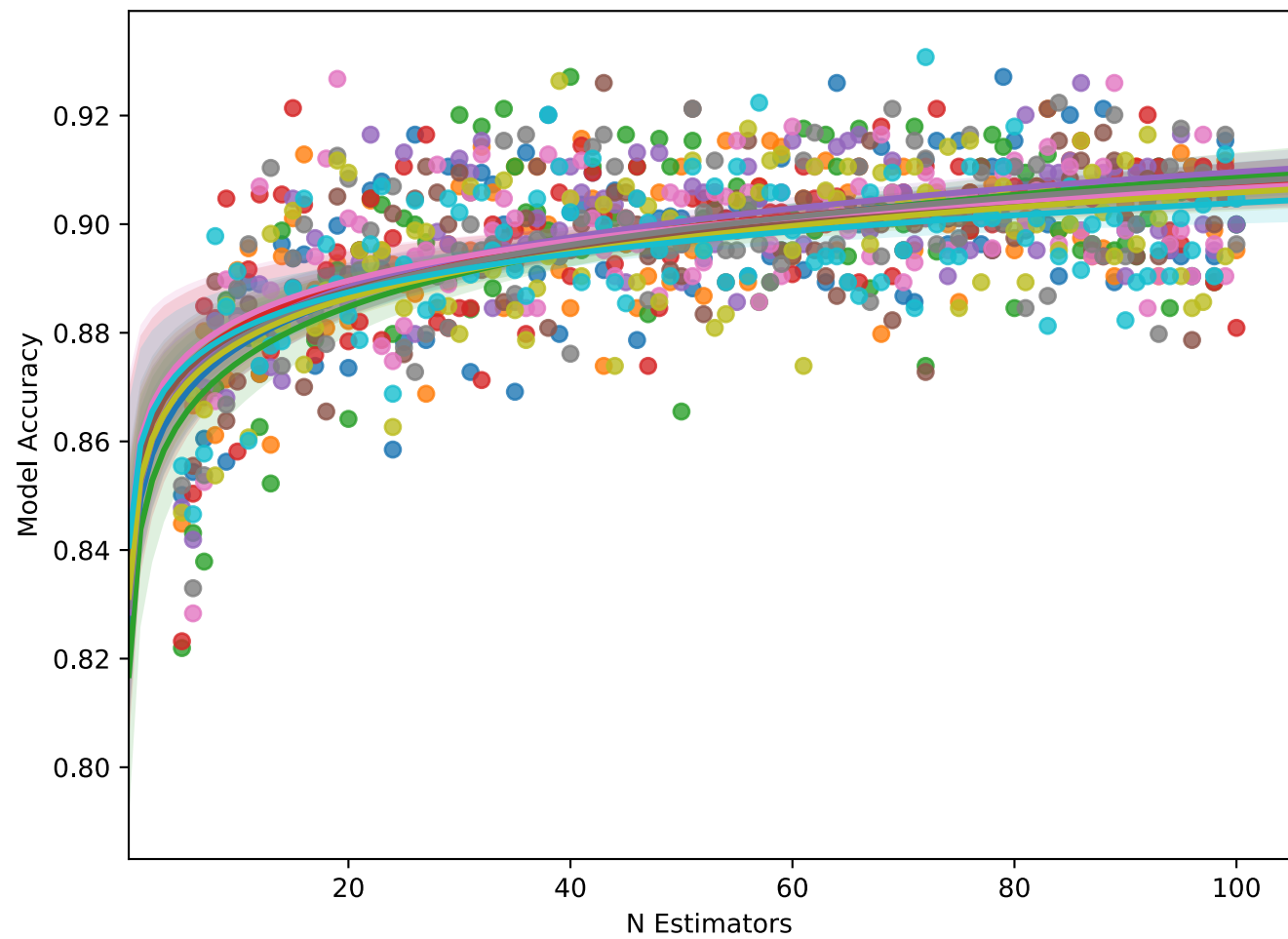

B

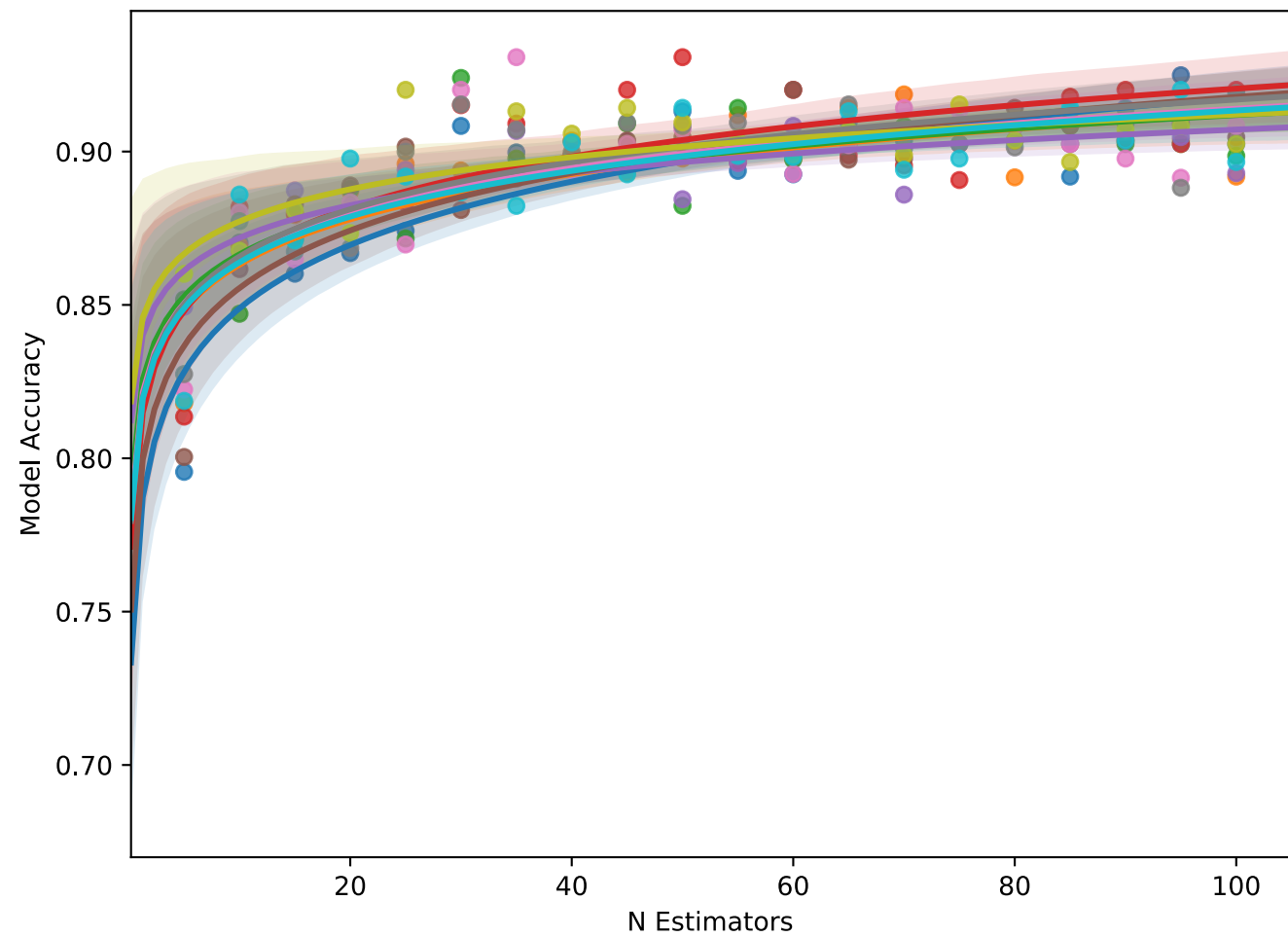

Supplement: Supplementary file 3 — Figure S3. Hyperparameter tuning for n_estimators in the assembly-based approach. Each figure shows accuracy results from a series of random decision tree constructions and random train/test partitions for each of those constructions. (A) Hyperparameter tuning of the random paired-end subset assembly (PP). (B). Hyperparameter tuning of the left-only assembly (PL). Note: The difference is point count is from fewer tests in the PL assembly as it had 10X as many features and took much longer to train and test. (PDF 2103 kb) [file 13062_2019_242_MOESM3_ESM.pdf]
